# A New Construct in Career Research: Career Crafting

**DOI:** 10.3390/bs13010049

**Published:** 2023-01-06

**Authors:** Xiaolin Ge, Lei Gao, Haibo Yu

**Affiliations:** School of Government, Beijing Normal University, Beijing 100875, China

**Keywords:** career crafting, job crafting, career self-management, proactive behaviors

## Abstract

Career crafting is a new concept in the field of career research in recent years. However, the research on career crafting is still in its infancy, and there are few systematic and integrated studies. In this study, we have collected the existing research and extracted the 12 most related articles from 10 databases (Web of Science, Google Scholar, ProQuest, and EBSCO Host, etc.) by the end of 2022 to discuss the concepts of discrimination, theoretical basis, research methods, and measurement tools and variables of career crafting. As a reference for the follow-up in-depth study, future research should progress forward, such as by deepening and expanding the theoretical basis, testing and developing mature scales, building multilevel influencing factors and testing their interaction, and furthering the research on the mechanism of multi-field effects.

## 1. Introduction

Crafting is not a new term in the field of career studies, and previous research can be found in the literature explaining career development [1,2,3]. In recent years, job crafting [4,5,6], which aims to enhance person–job fit through bottom-up job redesign, has become a hot research topic. In addition, career crafting takes the issue of how to better manage career development to a new level. Specifically, with the development of society, the original stable and idealized career development path has been impacted, resulting in a shift of individual perceptions from choosing a career to a wider and more fluid career planning process. At the same time, due to changes in individual needs, values, and abilities, the preferred job chosen at one time may not be the ideal job at another time. The abilities, skills, and knowledge required for a job may also change over time. Based on this reality, career crafting is gaining attention.

Initially, the concept most closely related to this review is Valcour’s [7] article in Harvard Business Review, which proposed “craft a sustainable career”. Next, Vidwans [8] proposed a new paradigm of career crafting based on qualitative research. Tims and Akkermans [6] and Lee et al. [9] further clarified the concept and developed measurement tools in their subsequent studies. To date, studies of career crafting are still relatively limited, and there is a lack of reviews. Research on career crafting is mainly focused on conceptualization and scale development, and the empirical research literature is scarce. Based on the frontier research on career crafting, we compose the relevant literature, discusses the conceptual analysis, theoretical foundation, research methods, and measurement tools and variables of career crafting, and provide an outlook on the future research direction of career crafting to provide a reference for subsequent research.

## 2. Concept and Identification

### 2.1. The Concept of Career Crafting

There are five main perspectives on the concept of career crafting. First, Valcour [7] proposes “craft a sustainable career”, arguing that the key is to understand oneself and adapt sharply to one’s field of interest and company and that crafting a sustainable career includes performing meaningful work, making full use of one’s skills, working with dynamic people, and being able to combine work with other important things, such as family, friends, and leisure life. Second, Vidwans [8] builds on the theory of job crafting with principles such as cognitive, task, and relationship crafting, further expanding on the external influences of family, organization, and environment while incorporating a gendered career development model and identifying a broader understanding of career success (personal cognitive and professional domains). Third, De Vos et al. [10] examines a new perspective on career choice, considering the changes that occur in the broader career context, emphasizing the dynamic and iterative nature of career choice, the central role of the individual in career behavior, and the importance of balancing personal and situational needs, and considering career crafting as “the new career choice”. They define career crafting as “the proactive behavior of individuals to optimize career outcomes by improving the personal-career fit” and to actively build their careers over time by reflecting on and focusing on their career aspirations and motivations to make choices that affect short- and long-term career success. Fourth, Tims and Akkermans [6] integrates the concepts of job crafting, career competencies, and career self-management, defining career crafting as “the proactive behavior of individuals to self-manage their careers, aiming to achieve the best personal-career fit”. Fifth, Lee et al. [9] emphasizes the integration of individual employee proactivity and congruence in a career environment, where proactivity is the ability of an individual to optimize resources outside oneself to achieve the desired job, while congruence refers to the alignment of an individual’s career with one’s internal interests, strengths, values, and needs. Career crafting is the proactive pursuit of congruence by individual employees who create or expand career-related resources based on the evolving nature of the job and explore career options that better align with their changing needs, values, and interests.

By comparison, Valcour [7] and De Vos et al. [10] proposes a definition based on reality in order to pursue career sustainability. This perspective on career crafting recognizes that individual needs and contextual demands are dynamic and that they can affect person–career fit at any given time [10]. Moreover, “crafting” in the field of careers has been utilized in the specifically related manner as used in job crafting theoretically [8]. Scholars expand the concept of job crafting to career crafting (e.g., [6,8,9]). Vidwans [8] validated career crafting paradigm expanded to family, organization, and environment. Different from this, Tims and Akkermans [6] and Lee et al. [9] pay more attention to other individuals’ behaviors, resources, and abilities, thus integrating other constructs, such as career self-management, career competencies, and career orientation.

### 2.2. Identification of Career Crafting and Related Concepts

To better understand the concept of career crafting and distinguish it from other similar concepts, this section selects the concepts borrowed and absorbed in the process of career crafting and compares career crafting with job crafting and career self-management.

#### 2.2.1. Career Crafting versus Job Crafting

Both career crafting and job crafting are the proactive behaviors of individual employees who aspire and strive to change their environment or themselves and are able to actively participate in crafting their future through the choices they make [9,11,12]. The differences between the two can be developed in the following ways.

In terms of the temporal dimension, job crafting examines how employees can change their current jobs and focuses on the short term. On the other hand, career crafting indicates that better adaptation to the present job and finding a new job based on changing needs, values, and interests should not be separated [9]. Career crafting is a long-term and cumulative process that encompasses the entire career path and involves the overall development of the career, not just the current job [8,13].

In terms of role behavior, job-level task crafting describes changing the type, scope, and number of job tasks; job-level relationship crafting refers to changing the quantity and quality of interactions with others at work [9]; and job-level cognitive crafting involves changing individuals’ perceptions of work, such as interpreting their work as part of their life story rather than a means of survival [5]. Career crafting also includes these three dimensions and is closely linked between them [9]. Career-level task crafting is defined as the extent to which individuals develop skills to achieve their best selves during their careers; career-level relational crafting is defined as the extent to which individuals actively seek out and connect with a group of people with whom they can share genuine interests and values; and career-level cognitive crafting is defined as the extent to which individuals actively reflect on the meaning of their careers and consider them to be an important and meaningful part of their lives [9].

In terms of research scenarios, job crafting focuses on events that occur in the workplace, with job-level variables selected to explore the impact of the work environment and job outcomes [8,14]. Career crafting also considers other important aspects, namely the long-term aspects of an individual’s career and life, such as family, organization, and external environment [8].

#### 2.2.2. Career Crafting versus Career Self-Management

Both career crafting and career self-management are important variables in the field of career development, and they have conceptual similarities as well as significant differences. Both of them are active career self-initiated and self-regulatory processes of individuals. However, career self-management refers to a problem-solving process by which individuals gather relevant information through career exploration and develop a greater awareness of themselves and their environment in order to develop career strategies [15]. Career crafting refers to the proactive behavior of individuals to self-manage their careers, aiming to achieve the best personal-career fit [6]. Career self-management emphasizes the development of a specific, predictable, and rational career goal through active efforts, while career crafting emphasizes the integration of the “proactivity” of seeking external resources and the “consistency” of internal interests, strengths, values, and needs [9] to pursue the dynamic matching process of personal will and career path rather than only pursuing rationalized career development goals. In addition, career self-management may be stimulated by both intrinsic motivation (e.g., pursuing personal interests and values) and extrinsic motivation (e.g., needing more income to survive) [16], so it pays attention to both. However, career crafting pays more attention to personal-career fit, emphasizing individual self-reflection and self-construction of a career, so it pays more attention to intrinsic motivation.

## 3. Theoretical Perspectives Adopted in Career Crafting

The current theoretical perspectives of career crafting research include the conservation of resources theory [17], career construction theory [18,19], and job crafting theory [4,5,6]. The first two are the theoretical foundations for empirical research on career crafting. The conservation of resources theory explores how individuals manage their resources to achieve their career goals through career crafting from a resource perspective; career construction theory explores individuals’ adaptive responses in career exploration through career crafting from a dynamic perspective; and job crafting theory is applied to the formulation of the concept of career crafting based on a role perspective.

### 3.1. Conservation of Resources Theory

Career crafting is viewed from a resource-based perspective as a proactive resource management behavior aimed at using, maintaining, and acquiring career resources to achieve career goals [20,21,22]. The conservation of resources theory explains the relationship between career success and career crafting as two competing motivations (i.e., resource conservation and resource acquisition), where both objective and subjective career success are resources for conducting career crafting. First, when individuals perceive low or high levels of available career resources, they are most strongly motivated to engage in resource acquisition and engage in active career reflection. The above theory explains the U-shaped relationship between subjective career success and active career reflection [23]. However, the cognitive element (i.e., active career reflection) generally precedes the behavior element (i.e., active career construction), and for those who lack career resources (i.e., low levels of subjective career success), engaging in active career reflection may deplete the resources needed to initiate subsequent autonomous goal-achieving actions, so only individuals with high levels of subjective career success are associated with active career construction behaviors [23]. Second, the conservation of resources theory suggests that individuals who lack resources are more vulnerable to resource loss, whereas individuals with more resources are more likely to gain further resources [24]. Therefore, more successful individuals are more willing to invest resources in career crafting, explaining the linear relationship between objective career success (number of promotions) and career crafting. Third, according to the complementarity problem in the conservation of resources theory [25], the importance individuals place on resources depends on the degree to which individuals perceive resources to help achieve their goals. Therefore, individuals who perceive a large number of learning opportunities at work are more likely to combine them with available resources (career success) for career crafting [23].

### 3.2. Career Construction Theory

The career construction theory [18,19] integrates the relationships of structural demands, adaptability resources, and adapting responses into a model. To begin with, according to career construction theory, the newly operationalized concept of career crafting [6] captures the aspects of career planning and career exploration, which can be seen as an adapting response of individuals [26]. Thus, the antecedents of career crafting could be researched based on CCT, such as how to transform career adaptability and competencies into this career adaptation response. Equally important, CCT suggests that differences in individual motivation for adaptation and adaptive behavior will lead to different adaptive outcomes in career development. It is also worthwhile to continue to understand the mechanism of the role of career crafting in career adaptation outcomes, as well as the influence of contextual factors. A present study investigating the impact of career adaptability on career crafting [26] found that career crafting can be predicted by an increase in one’s perceived structural needs, combined with the increased availability of adaptive resources, contributing to an understanding of the role of resources.

### 3.3. Job Crafting Theory

Job crafting theory has played an important role in the formulation and development of this concept. Career crafting and job crafting are similar, utilizing career-level task, cognitive, and relational crafting [8,9] to balance resources and demands in a career in an effort to achieve a personal career match. When the concept of career crafting was introduced, Vidwans [8] built on the theory of job crafting principles, such as cognitive, task, and relationship crafting, expanding it and incorporating elements of family, organization, and environment. In later studies, Tims and Akkermans [27] and Lee et al. [9] also developed the construct by replacing the word “job” with “career” based on the literature and scales related to job crafting. The measure of this construct was developed.

## 4. Research Methods of Career Crafting

Through a literature overview, we have determined that there are three main research methods used in career crafting. First, the questionnaire method, as a typical empirical research method, was used by Janssen et al. [23] and Nalis et al. [26], who distributed and collected questionnaires with a sample of 702 teachers and 2000 people working at Gelman, respectively, and tested the proposed hypotheses through structural equation modeling (SEM).

The second method is experimental intervention. The career crafting study used an intervention mapping program in a systematic way, such as identifying needs, developing the program, pilot testing, implementing, and evaluating [28]. Specifically, the study participants were divided into experimental and control groups, and the experimental group underwent a four-hour training session on career crafting and was asked to work on self-imposed goals afterward, during which coaching calls were conducted, and post-intervention outcome variables were assessed by questionnaire after 8 weeks.

The third method for career crafting research is qualitative research. Career crafting was conceptually developed when one study conducted semi-structured in-depth interviews with 36 accounting professionals in New Zealand and compiled a total of approximately 170,000 words of recorded text. The data were subjected to NVivo, extraction of key events, and coding [8]. The follow-up study continued to focus on outstanding female accountants of different nationalities, using methods such as interviews and coding to address how gender factors combined with family and organizational contextual circumstances are reflected in the concept of career crafting [29,30].

To sum up, the questionnaire method has the advantages of high efficiency, good objectivity, and easy promotion. However, the development of career crafting scales is limited at present, and the effectiveness and application situation of the existing scales need to be further tested. In addition, the questionnaire method is a cross-sectional study, which cannot test the cause and effect. The experimental intervention can test the causality, but it is greatly affected by the experimental operator, and it is difficult to completely exclude the influence of interference variables. Qualitative research is more flexible, can deeply explore the real ideas of the interviewees, and is suitable for the exploration of antecedents and mechanisms. However, it requires a high level of data induction and a theoretical level of the researchers. Future research should choose appropriate research methods according to research situations and research purposes. Considering the dynamics and complexity of career crafting, future research on career crafting can adopt longitudinal research design to track individuals for a long time, and cross-level research to explore the more complex antecedents and outcomes of career crafting.

## 5. Measurement of Career Crafting

There are two main career crafting scales currently being developed. Tims and Akkermans [27] originally developed the career crafting scale and tested it in a Western context. Based on a review of the literature on job crafting, career competence, and career self-management, they developed a six-point career crafting scale with two dimensions and eight questions based on existing questions. The scale consists of two dimensions: proactive career reflection and proactive career construction. First, proactive career reflection refers to proactive behaviors that focus on exploring and assessing career-related motivations, values, and goals. This dimension is related to concepts such as cognitive job crafting [5], cognitive career self-management [31], and reflective career competence [32]. Second, positive career construction includes career-related interpersonal relationships, self-description (self-profiling), and goal-striving proactive behaviors. This dimension builds on the concepts of structural and social job crafting [33], career self-management [31], and communication and career competence [32]. The scale has good reliability and validity, with an internal consistency coefficient of 0.80 for the active career reflection dimension and 0.85 for the active career construct dimension.

Using a deductive approach, Lee et al. [9] combined the Job Crafting Scale [34], the Job Crafting Scale based on the Job Demands–Resources Model [33], the Career Planning Scale [35], the Career Self-Management Scale [36], the Focus on Internal Career Characteristics Scale [37], the Authenticity, Balance, and Challenge Scale [38], and the Changing Career Attitudes Scale [39] to develop a 4-dimensional, 15-question scale with 7 different scales. The reliability and validity of the scale was good, with internal consistency coefficients of 0.93 for the changing relational boundaries dimension, 0.86 for the utilizing relational resources dimension, 0.91 for the reflecting positive career meaning dimension, and 0.90 for the expanding task boundaries dimension. The internal consistency coefficient of the overall questionnaire was 0.93.

Both of these scales were developed based on job crafting and existing career-related research, but there were significant differences between the two scales. Specifically, (1) the two dimensions of the scale developed by Tims and Akkermans [27] are more relevant to the conceptual context of career crafting, and both dimensions point to the self. The process of individuals actively adjusting their career development by continuously examining their inner selves is precisely the process of active career reflection and active career construction, and contextual mining from the original concept eventually leads to the measurement instrument. (2) The dimensions of the career crafting scale developed by Lee et al. [9] are more specific, and the instrument is developed based on existing scales related to the concepts defined by the authors. All four dimensions point to the external world (tasks, jobs, etc.).

## 6. Review of Variables Related to Career Crafting

**Figure 1:**
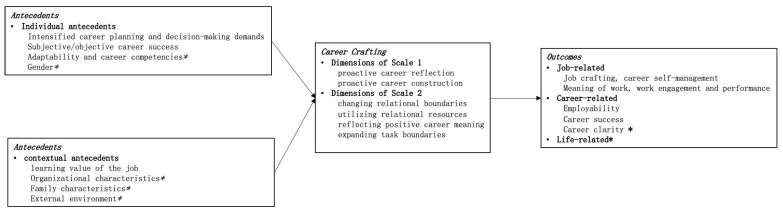
Diagram of the theoretical framework of career crafting (summary of this study, * represents empirical tests in the future).

The following section maps the nomological network of variables to which career crafting is related. Specifically, it unpacks the antecedents (individual and contextual) and outcomes (Work-related, Career-related, and Life-related) of career crafting (see Figure 1). We classified the antecedents and consequences in the following sections according to the perspectives adopted in each manuscript, regardless of whether the measurement was cross-sectional, correlational, or longitudinal.

### 6.1. Antecedents

#### 6.1.1. Individual Antecedents

Empirical studies of individual factors have demonstrated the relationship between intensified career planning and decision-making demands, subjective/objective career success, and career crafting; in addition, factors such as adaptability, career competencies, and gender have been proposed based on the concept of career crafting.

Intensified career planning and decision-making demands. Individuals are the strategists and implementers of their own careers, which means that they face the need to plan their careers and make career decisions [40]. As this individual need increases, it stimulates a career-adapting response (e.g., career crafting). An empirical study verified the above hypothesis that individuals with highly intensified career planning and decision-making demands show a positive relationship with the dimensions of career crafting (i.e., active career reflection and positive career construction) [26].

Subjective/objective career success. Janssen et al. [23] demonstrated that subjective career success predicting the two dimensions of career crafting was not all linearly related. For example, subjective career success had only a U-shaped relationship with active career reflection, and only higher levels of subjective career success were associated with higher levels of active career construction (i.e., strengthening the secondary relationship). In contrast, objective career success (i.e., the number of career promotions as a whole) is positively and linearly related to both dimensions of career crafting.

Adaptability and career competencies. Theoretical analysis suggests that adaptability and career competencies, as individual resources and competencies, are two important factors in career crafting [10]. Career adaptability is an important foundation for career agency and employability. Career-competent employees who meet the needs of the present and the future are expected to better protect and promote career sustainability through career crafting [27]. Researchers have also examined the interaction of adaptability with individual career planning and career decision-making needs on career crafting [26]. Akkermans and Tims’ [41] findings show that career competencies may act as a personal resource that can trigger a motivational process associated with establishing a healthy work–home balance via expansive job crafting when crafting your career.

Gender. It is generally believed that women are more likely than men to make employment concessions for family responsibilities and women more often explain success from a family perspective. Vidwans [8,29,30] has emphasized the influence of gender factors on individuals’ career crafting in qualitative studies. Through the analysis of interview data and the reports of female accountants, it was found that women can achieve a balance of multiple selves, such as the career self, the possible self, the spouse self, and the mother self, through cognitive crafting, relationship crafting, and task crafting and break the exclusive gender barriers to achieve success in combination with the contextual environment of the family and the organization.

#### 6.1.2. Contextual Antecedents

Contextual factors include the learning value of the job and the influence of organizational characteristics, family characteristics, and the external environment.

Learning value of the job. Janssen et al. [23] used perceived learning value in the job context as a moderating variable and found that this variable reinforced the relationship between subjective career success and the dimensions of career crafting. This is because when individuals perceive that the job has high levels of learning value (e.g., learning and development opportunities, etc.), combined with psychological resources, such as perceived career success, individuals will feel a sense of progress and growth, which promotes career crafting as a resource acquisition behavior.

Organizational characteristics. The dynamic interaction between individuals and organizations can affect the person-career fit at any time [10]. Organizations influence individuals’ careers by providing learning opportunities, supporting employees in developing career competencies, helping employees maintain employability, and engaging in career conversations about employees’ current positions and future perspectives, all of which facilitate career choices that help craft sustainable careers [42,43,44]. In addition, supervisors may have an influence as they support individuals in achieving career goals or may be seen as role models of how to manage a career [27].

Family characteristics. Theoretical analysis suggests that two aspects of family life may have an impact on career decisions. One is family of orientation, which refers to a person’s birth family, such as parental emotional and financial support and family environmental factors that influence the early career development of children and adolescents; the other is family of procreation, which refers to the family an individual establishes with a life partner [45]. Research has shown that spousal support can provide resources for effectively managing one’s career and that family responsibility and support have a significant impact on an individual’s career crafting [8].

External environment. Vidwans [8] suggested that external factors such as social, cultural, political, legal, technological, natural, and global factors can influence individual career crafting. Although these factors are beyond the control of the individual, career crafting focuses on the individual’s response to environmental changes. Furthermore, Tims and Akkermans [27] also indicated that studying how the social environment influences (facilitates or inhibits) career crafting and its outcomes would be the way forward.

### 6.2. Outcomes

#### 6.2.1. Work-Related Outcomes

Prior work on the outcomes of career crafting has focused on a broad range of outcomes, including job crafting, meaning of work, work engagement, and performance, which are empirically drawn.

Job crafting, career self-management. The results of an intervention study [28] showed that the career crafting intervention enhanced individuals’ perceptions of career self-management and job crafting and reduced barriers to work demands.

Meaning of work, work engagement, and performance. Some theoretical analyses suggest that career crafting is an active behavior of individuals and involves choices that affect both short-term (e.g., work engagement and performance) and long-term success (e.g., objective and subjective career success) through reflection on career ambitions and motivations [10]. Lee et al. [9] suggest that career crafting predicts the meaning of work and work engagement while also explaining the unique differences outside of job crafting. In turn, work engagement predicts higher job performance [46], implying a strong relationship between career crafting and work engagement.

#### 6.2.2. Career-Related Outcomes

The main outcome variables at the career level, such as career success, employability, and career clarity, were also validated in the qualitative study. They are developed as follows.

Employability. Regression results from a study [27] concluded that while controlling for organizational career management, both dimensions of career crafting were positively associated with internal employability (i.e., current employer), while only active career reflection was positively associated with external employability (i.e., other employers). However, there was no evidence of an impact of the career crafting intervention on employability. Leeuwen et al. [28] explained that since employability refers to the future, this fact is affected by the so-called “sleeper effect”, which may make it difficult for the participants to perceive changes in their employability within 8 weeks after the intervention.

Career success. Researchers [9] concluded that career crafting predicts career success, while further regression showed that career crafting explained more variance than job crafting in terms of work meaningfulness and work engagement but not in terms of subjective career success. This result suggests that even if career crafters are satisfied with their current jobs, they may still want to find more engaging and meaningful work in their careers. De Vos et al. [10] also suggest that career crafting is a proactive behavior that affects subjective and objective career success.

Career clarity. Career crafting (including cognitive, task, and relationship crafting) plays a central role in gaining career clarity. Analysis of interview data found that career crafters who received support from their family and school performed exceptionally well in terms of their clarity of purpose and career path progression and that career crafting can transform individuals who initially had low levels of career clarity into individuals who currently have high levels of career clarity [8].

#### 6.2.3. Life-Related Outcomes

The impact of career crafting on life aspects is currently mainly based on theoretical analysis. Vidwans [8] established that career crafting leads to success in personal domains, such as maintaining physical health, spending time with children, fulfilling family responsibilities, participating in community activities, spending time with friends, achieving financial stability, and pursuing personal interests, among other markers. There are also studies [29] in which the analysis of the career crafting process of female accountants demonstrated that individual commitment to career and family is not irreconcilable but can be skillfully combined.

## 7. Agenda for Future Research on Career Crafting

**Figure 2:**
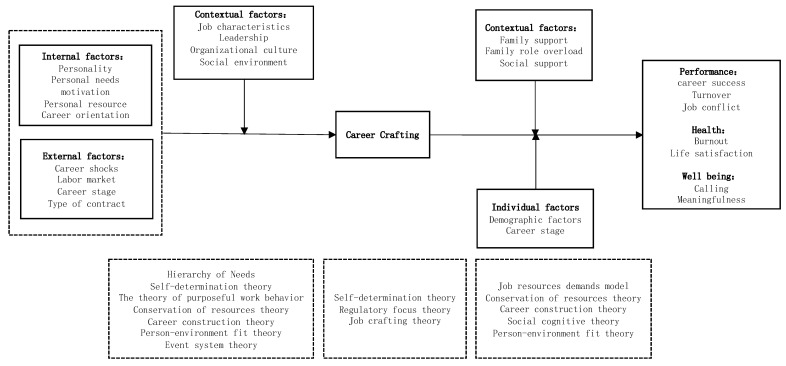
A model of career crafting with a testable proposition.

Based on previous studies, it is clear that more work needs to be performed to understand career crafting, which is a new construct of career research. Our review highlighted significant gaps in our knowledge and weaknesses in the approaches adopted in previous work. Below, we outline a detailed agenda for future research on career crafting that targets opportunities for theoretical and empirical advancement of the field. We attempt to propose an integration framework for future research, as shown in Figure 2.

### 7.1. Expanding the Theoretical Basis of Career Crafting

The existing theoretical bases have their own focus and shortcomings in relation to the concept of career crafting, which is currently in the development stage. First, resource conservation theory views career crafting as a process of gaining/losing resources and explains career crafting from a motivational perspective. The shortcomings are that the study selected only the perceived learning value of work as a moderating variable and did not consider the influence of other external environments, such as family, leadership, and social factors; the cross-sectional research design inhibits the ability to draw causal conclusions that resources may also influence the achievement of career success because acquired and mobilized career resources may contribute to further career success (i.e., reverse causality) [23]. Second, career construction theory, which explains career crafting as career-adaptive behavior focusing on active adjustment and choice behavior during the career and has been tested in empirical studies but ignores the cognitive crafting of individuals, is better suited to explain the process and nature of career crafting in the temporal dimension. Third, job crafting theory provides a framework integration of tasks, relationships, cognitive crafting, and the external environment based on job roles [8,9], which is the conceptual basis of career crafting but does not sufficiently consider the endogenous motivation of individuals to engage in career crafting.

Based on the above theoretical bases, the theoretical bases of career crafting research can be further deepened and expanded in the future. First, we can provide explanations for more situations from multiple theoretical perspectives, such as interpreting the concept using the process of individual self-determination based on self-determination theory and studying the connotation of career crafting from two kinds of motivations, namely, promotion-orientation and prevention-orientation, based on regulatory focus theory. Second, we expand the theoretical research of the concept of career crafting, combine many different situations, provide theoretical guidance for the current practice of individual employees and organizations, and explore the connotation and extension of career crafting in different groups, industries, and situations to make sufficient theoretical accumulation for practical application.

### 7.2. Testing and Developing the Measurement of Career Crafting

The development of career crafting scales is still at an initial stage. Only Tims and Akkermans [27] and Lee et al. [9] have developed relevant measurement tools, but these scales have been developed for a short period of time, and their reliability and applicability contexts need further validation and analysis. Future research can be conducted in several ways. First, the two scales differ significantly in terms of dimensions and specific questions, and the rationale and process of scale development also differ significantly. In the future, the two scales with different characteristics can be further integrated to obtain a measurement tool with better reliability and validity. Second, the development of both scales is based on Western management contexts and lacks analysis and consideration of different countries. Third, we can further investigate the influencing factors and mechanisms of career crafting through qualitative research methods and develop a new measurement combined with the questionnaire method and other methods.

### 7.3. Improving Empirical Studies of Career Crafting

#### 7.3.1. Constructing Multilevel Antecedents and Examining Their Interactions

The current research mainly proposes hypotheses based on job crafting, and less empirical research has been carried out. The influencing factors of career crafting can be explored from multiple perspectives based on different theoretical foundations. First, from the perspective of personality, empirical studies can be conducted to examine whether a proactive personality, core self-evaluation, and the big five personalities influence this behavior. From a motivational perspective, based on the hierarchy of needs theory, the self-determination theory, and the purposeful work behavior theory, what kinds of individual needs and high-order goals promote employees’ career crafting? Personal resources such as self-efficacy, career resilience, and adaptability are more likely to craft one’s career and to trigger individuals to adopt career crafting based on the conservation of resource theory and career construction theory. Secondly, event system theory (EST) [47] emphasizes the important role played by events in triggering organizational action and changes in organizational characteristics [47]. Akkermans et al. [48] think it provides an elaborate theoretical framework for studying the individual responses given to career-related events. Therefore, career shocks may be considered to understand career crafting [27]. The labor market, career stage, and type of contract may also be external antecedents. As van den Groenendaal et al. [16] mentioned, the characteristics of the contemporary labor market are the rapid growth of the number of solo self-employed who lack valuable career resources. They must find other ways to take full responsibility for their own work and establish a sustainable career. Therefore, future research may focus on how the solo self-employed can craft their career. A longitudinal research design can be used to incorporate time into the research framework considering the dynamic and complex nature of career development [10,23] and to track the influencing factors of individuals’ career-crafting behaviors over a longer period of time. Thirdly, from the organizational characteristics in terms of person-environment fit theory, individuals belong to different teams or organizations, so leaders will be an important contextual factor for employee crafting. Future research should combine leadership and career crafting, focusing on the direct impact of different leadership styles on career crafting and how to motivate employees’ career crafting. The exploration of the influencing factors of variables such as job characteristics and organizational culture also needs to be further expanded to study the influence of different factors of the organization on career crafting using cross-level studies. Additionally, whether the social environment and major social events such as the COVID-19 epidemic have an impact on individuals’ career crafting can be explored causally by tracking.

#### 7.3.2. Deepening Research on the Effects of Career Crafting in Multiple Domains

More empirical studies are still needed to explore the outcomes of career crafting. First, the outcome variables of existing work domains are mostly focused on the positive effects, such as career success, and future research can expand the negative effects of career crafting and explore the relationship between career crafting and turnover and job conflict. Second, the outcomes are studied from different theoretical perspectives. Combining different theories, such as job resources demands model, conservation of resources theory, and career construction theory, more research should be conducted to explore the relationship between career crafting behavior and more non-career domain variables, for instance, health, such as burnout and life satisfaction, and eudaimonic well-being, such as calling and meaningfulness. Third, different moderator variables are sought to explore the mechanisms of career crafting. Demographic factors such as gender, career stage, income, age, and socioeconomic status and career stage can be used to influence the mechanism of career crafting to different degrees, and some family-level factors, such as family support, family role overload, and social support from supervisors and colleagues, can be considered to be introduced in relation to career crafting based on social cognitive theory and person-environment fit theory.

## 8. Conclusions

The purpose of this review was six-fold. First, we discussed how career crafting has been conceptualized in previous research and elaborated on the evolution and the conceptually difference from related constructs such as job crafting and career self- management. Second, we reviewed the theoretical perspectives that have been used in prior research. Third, we listed the research methods used in the present studies. Fourth, we laid out the measurement tools and differences between the two scales. Fifth, we mapped the theoretical framework of variables to which career crafting is related by reviewing work on its antecedents and outcomes. Finally, we proposed a future research agenda for career crafting that targets opportunities for empirical and theoretical advancement of the literature. We hope this review provides a basis from which future research can be developed. 

## Data Availability

All of the data for this study will be made available upon reasonable request.
